# Post-resuscitation shock: recent advances in pathophysiology and treatment

**DOI:** 10.1186/s13613-020-00788-z

**Published:** 2020-12-14

**Authors:** Mathieu Jozwiak, Wulfran Bougouin, Guillaume Geri, David Grimaldi, Alain Cariou

**Affiliations:** 1 Service de Médecine Intensive Réanimation, Hôpitaux Universitaires Paris-Centre, Hôpital Cochin, Assistance Publique-Hôpitaux de Paris, 27, rue du faubourg Saint Jacques, 75014 Paris, France; 2 Université de Paris, Paris, France; 3 Service de Médecine Intensive Réanimation, Hôpital Privé Jacques Cartier, Ramsay Générale de Santé, Massy, France; 4 INSERM U970, Paris-Cardiovascular-Research-Center, Paris, France; 5 Paris Sudden-Death-Expertise-Centre, Paris, France; 6 Service de Médecine Intensive Réanimation, Hôpital Universitaire Ambroise Paré, Assistance Publique-Hôpitaux de Paris, Boulogne-Billancourt, France; 7 Université Paris-Saclay, Paris, France; 8 INSERM UMR1018, Centre de Recherche en Epidémiologie Et Santé Des Populations, Villejuif, France; 9 Service de Soins Intensifs CUB-Erasme, Université Libre de Bruxelles (ULB), Bruxelles, Belgium; 10 AfterROSC Network Group, Paris, France

**Keywords:** Cardiac arrest, Coronary angiogram, Ischemia–reperfusion syndrome, Mean arterial pressure, Myocardial dysfunction, Targeted temperature management, Vasopressors

## Abstract

A post-resuscitation shock occurs in 50–70% of patients who had a cardiac arrest. It is an early and transient complication of the post-resuscitation phase, which frequently leads to multiple-organ failure and high mortality. The pathophysiology of post-resuscitation shock is complex and results from the whole-body ischemia–reperfusion process provoked by the sequence of circulatory arrest, resuscitation manoeuvers and return of spontaneous circulation, combining a myocardial dysfunction and sepsis features, such as vasoplegia, hypovolemia and endothelial dysfunction. Similarly to septic shock, the hemodynamic management of post-resuscitation shock is based on an early and aggressive hemodynamic management, including fluid administration, vasopressors and/or inotropes. Norepinephrine should be considered as the first-line vasopressor in order to avoid arrhythmogenic effects of other catecholamines and dobutamine is the most established inotrope in this situation. Importantly, the optimal mean arterial pressure target during the post-resuscitation shock still remains unknown and may probably vary according to patients. Mechanical circulatory support by extracorporeal membrane oxygenation can be necessary in the most severe patients, when the neurological prognosis is assumed to be favourable. Other symptomatic treatments include protective lung ventilation with a target of normoxia and normocapnia and targeted temperature management by avoiding the lowest temperature targets. Early coronary angiogram and coronary reperfusion must be considered in ST-elevation myocardial infarction (STEMI) patients with preserved neurological prognosis although the timing of coronary angiogram in non-STEMI patients is still a matter of debate. Further clinical research is needed in order to explore new therapeutic opportunities regarding inflammatory, hormonal and vascular dysfunction.

## The concept of post-resuscitation shock

Outcome of cardiac arrest (CA) remains very poor. Over 60% of patients with out-of-hospital cardiac arrest (OHCA) will die without sustainable return of spontaneous circulation (ROSC) [1]. Among patients with sustainable ROSC, intensive care unit (ICU) mortality remains high, ranging from 60% [2, 3] to 80% [1, 4] of patients. In-hospital mortality after OHCA mainly results from different causes including recurrent CA, irreversible anoxic brain damage (including brain death), as well as comorbid withdrawal of care [5, 6]. In addition, a substantial proportion of these post-CA patients will suffer from a severe hemodynamic impairment that may worsen organ damages and may lead to death in the first hours or days. All these complications are closely related to the duration of no-flow and low-flow of CA and thus to the severity of the oxygen debt of the different organs.

The first description of this post-resuscitation shock was provided by Vladimir Negovsky more than 45 years ago [7]. In a series of animal experiments and clinical observations, he has reported a myriad of clinical and biological changes that could be observed as a consequence of the whole-body ischemia and reperfusion provoked by CA. Among these disorders, the hemodynamic impairment is one of the most frequent and most severe alterations. Today, it is commonly accepted that after resuscitation and ROSC, the combination of tissue hypoperfusion and arterial hypotension requiring a continuous infusion of vasopressor may correspond to the most pragmatic definition of this shock [3, 8, 9]. Using this definition, the incidence of the post-resuscitation shock ranges between 50 and 70% [3, 8, 9]. In a retrospective cohort of patients admitted in ICU after an OHCA, Lemiale et al. reported a global incidence of 68%, and they also identified some factors (male gender, shockable rhythm, time to ROSC) associated with its occurrence [3].

In-hospital mortality attributable to this post-resuscitation shock varies between 20 and 55% [2, 3, 5] and most often results from multiple-organ failure, including (1) myocardial dysfunction in up to two third of patients [10], (2) acute renal failure in 10–80% of patients according to the definition used with a pooled incidence of 37% [11], requiring renal replacement therapy in one third of patients [12] and associated with long-term occurrence of chronic kidney disease [13], (3) hypoxic hepatitis in almost 15% of patients [14, 15] and (4) metabolic acidosis in up to 90% of patients [16]. All these organ failures were shown to be associated with poor outcome in this setting.

In this review, we aimed at focusing on the recent advances in pathophysiology and treatment of post-resuscitation shock.

## Pathophysiology of post-resuscitation shock

The pathophysiology of post-resuscitation shock is both due to the cause of CA and to the ischemia–reperfusion syndrome, which results in a complex and multifactorial puzzle of organ dysfunctions. Whatever the aetiology of CA, the post-resuscitation shock is mainly a combination of myocardial dysfunction, vasoplegia and hypovolemia.

### Myocardial dysfunction

In a pivotal study combining angiographic data and pulmonary artery catheter monitoring, Laurent and colleagues prospectively described the hemodynamic profile of consecutive patients after CA from cardiac origin before ICU admission at the time of initial left ventricular angiography and within the first 72 h of ICU stay [17]. When left ventricular angiography was performed, the ejection fraction was reduced in all patients, whereas filling pressure was increased in patients with hemodynamic instability but low to normal in patients without hemodynamic instability. Few hours after ICU admission, the cardiac index was found to be decreased with low or normal filling pressure in all patients, suggesting hypovolemia. Thereafter, the cardiac index gradually improved with a return to normal values within 24 h, whereas filling pressure remained unchanged over time. Despite improvement of cardiac index, all patients required large amount of fluid administration and high doses of vasopressors within the first 72 h to maintain acceptable mean arterial pressure (MAP) level [17]. On the whole, these observations were suggestive of an early and severe myocardial dysfunction, usually regressive within 48 h, associated with a vasoplegia.

Using echocardiography, it has been shown that this post-resuscitation myocardial dysfunction is very common, concerning up to 70% of the patients [10, 18]. The most common pattern is an early and transient systolic and diastolic left ventricular dysfunction [17], which can be considered as a model of myocardial stunning following the ischemia–reperfusion syndrome [19]. Of course, this myocardial dysfunction is very common when CA results from a coronary occlusion. However, this myocardial dysfunction may be worsened by repeated defibrillations (especially when using a monophasic and high-energy current), and may also be partly considered as an “adrenergic cardiopathy”, as illustrated by the independent association between the epinephrine dose administered during cardiopulmonary resuscitation and the severity of the cardiac dysfunction [20].

### Vasoplegia

Regarding the vasoplegia that is commonly observed as a consequence of the ischemia–reperfusion syndrome, two mechanisms are mainly suspected on the basis on prospective human studies. First, neutrophils accumulation, neutrophil-endothelial interaction and neutrophils activation in microvessels following global ischemia and reperfusion lead to endothelial cell dysfunction [21]. The latter increases the transduction of inducible NO-synthase, which in turn induces a relaxation of vascular smooth muscle cells and promotes the activation of the coagulation cascade [22]. Second, the reactive oxygen species generated by the ischemia–reperfusion syndrome activate the innate immune cells. It leads to an increase in inflammatory cytokines release and inducible NO-synthase expression, both worsening the endothelial dysfunction and thus vasoplegia [23, 24]. Since these two mechanisms are very similar to those involved in the pathophysiology of sepsis, the post-resuscitation shock is frequently considered as “a sepsis-like syndrome”. In addition, an authentic sepsis may also contribute to this hemodynamic profile as infectious complications are very common at this stage [25].

### Hypovolemia

Hypovolemia after CA is common but often undertreated, because of the fear of fluid overload in these patients with potential myocardial dysfunction. It results from vasoplegia (relative hypovolemia due to the mismatch between contents and container), from the capillary-leak syndrome in the most severe patients with prolonged resuscitation before ROSC and/or under extracorporeal life support (ECLS) [26] and in a later phase, from the third compartment syndrome related to ileus and intestinal injury.

### Splanchnic dysfunction and endotoxemia

Some human and prospective studies have suggested that gut injury could also contribute to the vasoplegia observed in the post-resuscitation shock through its ability to provoke or worsen a systemic inflammatory response [27–29]. Indeed, in post-CA patients, markers of intestinal injury are increased and endotoxemia is frequent [27, 28], this latter being associated with the severity of vasoplegia [29]. However, its incidence is still unknown and the relationship between post-resuscitation shock and gut injury is complex, as the two are closely intertwined.

### Hormonal dysfunction

A relative adrenal insufficiency could also participate to the vasoplegia observed during the post-resuscitation shock. Pène and colleagues prospectively performed corticotropin-stimulation test in consecutive post-CA patients admitted in ICU and they observed that 52% of these patients had a relative adrenal insufficiency that was associated with shock-related mortality [30]. These findings were confirmed by other teams [31, 32], highlighting the concept of both relative adrenal insufficiency and adrenal reserve exhaustion (as observed in patients with septic shock). In addition, the hypothalamic release of arginine-vasopressin seems to be impaired in patients after CA [33], also contributing to vasoplegia.

**Fig. 1:**
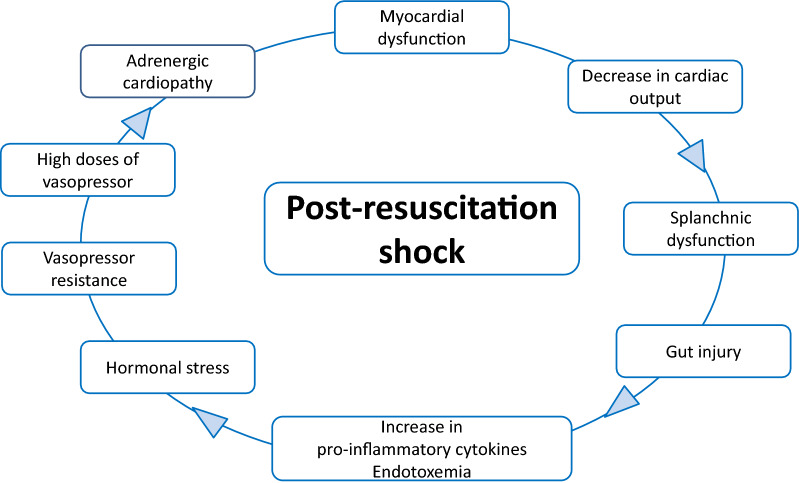
Vicious circle perpetuating post-resuscitation shock

Importantly, there is an interindividual variability in the respective weight of the different mechanisms described above in the pathophysiology of post-resuscitation shock. Nevertheless, all are closely interplayed and result in a vicious circle that self-perpetuates it (Fig. 1).

## Management of post-resuscitation shock

### Symptomatic treatments

#### Early-goal directed therapy

**Fig. 2:**
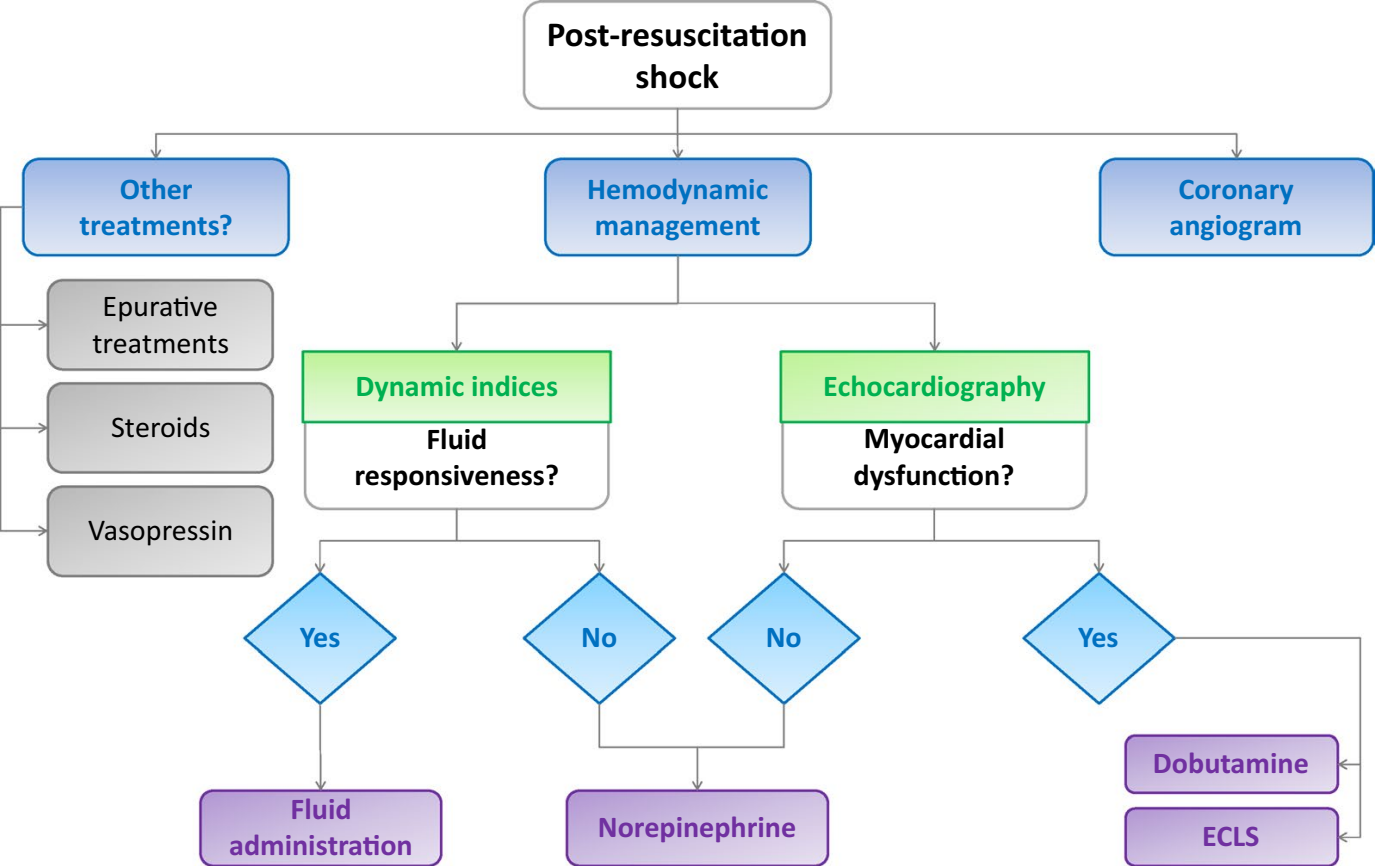
Proposal for management of post-resuscitation shock. *ECLS* extracorporeal life support

Similarities between septic and post-resuscitation shock led some authors to advocate for post-resuscitation shock an early-goal directed therapy strategy including hemodynamic resuscitation and therapeutic hypothermia [34, 35]. The hemodynamic resuscitation that is proposed is based on an aggressive step-by-step strategy including fluids, vasopressors, inotropes and blood transfusion, in order to target predefined MAP level and to normalize the central venous oxygen saturation, used as a surrogate of oxygen delivery within the first hours of therapy. Preliminary results from an exploratory study including 20 patients without systematic assessment of cardiac function suggested that this early-goal directed therapy did not improve mortality after comparison with matched historic controls [34]. A recent multicentre and randomized study confirmed that such an early-goal directed therapy strategy was neither shown to improve mortality nor to limit the extent of anoxic brain damage or neurological outcome despite an improvement in cerebral oxygenation [35]. Nevertheless, it might be reasonable to use such a strategy in post-resuscitation shock in order to maintain an adequate organ perfusion (Fig. 2).

#### Vasopressors and inotropic drugs

Besides fluid administration, the hemodynamic management of post-resuscitation shock is mostly based on vasopressors because of the severe vasoplegia and vasodilation, in combination with inotropes when post-resuscitation myocardial dysfunction is present (Fig. 2).

Norepinephrine should be considered as the first-line vasopressor, in order to avoid arrhythmogenic effects of other catecholamines. Regarding inotropes, dobutamine is the most established treatment in this situation [36, 37]. These two animal studies showed that dobutamine successfully overcome the global systolic and diastolic left ventricular dysfunction resulting from prolonged CA [36, 37]. In addition, the most effective dose would be 5 µg/kg/min: a lower dose would be inefficient and a higher dose would increase in a too large extent the myocardial oxygen consumption [37]. Importantly, this threshold value of 5 µg/kg/min could not necessarily be transposed in humans and the potential detrimental effects of higher dose of dobutamine deserve further studies.

Levosimandan could also be an interesting alternative to dobutamine in this setting, as suggested by an animal study [38], as well as phosphodiesterase inhibitor such as milrinone [20]. Nevertheless, nothing was done in this field since nearly one decade and both treatments still require further clinical validation and are not recommended for the management of post-resuscitation shock so far.

#### Target for mean arterial pressure level

Because arterial hypotension is associated with poor neurological outcomes in patients after CA [39] and because the autoregulation of cerebral blood flow may be impaired after ROSC [40], the MAP level is an important therapeutic goal in patients with post-resuscitation shock. In this regard, several observational studies have suggested that maintenance of higher MAP levels was associated with a better brain tissue oxygenation [41], an improvement in survival [42, 43] and a better neurological outcome [44]. In a recent multicenter and randomized study (COMACARE study), it has been shown in 120 comatose patients after OHCA that, targeting a low-normal (65–75 mmHg) or a high-normal (80–100 mmHg) MAP level for the first 36 h after ICU admission neither affect the neuron-specific enolase (NSE) serum level nor mortality or neurological outcomes [45]. However, targeting a high-normal MAP level was recently shown to decrease troponin release as a marker of myocardial injury [46]. Thus, although it is currently recommended that hemodynamic treatments should be guided by arterial pressure, the optimal MAP level still remains unknown and may probably vary according to patients [47].

#### Ventilatory management

A vast majority of post-CA patients are mechanically ventilated, according to current guidelines [47]. Regarding protective lung ventilation strategies, it has been shown in a retrospective study that lower tidal volumes (≤ 6 mL/kg) were independently associated with favorable neurocognitive outcome, more ventilator-free days and more shock-free days [48]. Regarding the positive end-expiratory pressure (PEEP) level, a secondary analysis of three international prospective, observational and multicenter studies including 812 patients mechanically ventilated after CA, showed that the PEEP level increased from 3.5 ± 3 to 6.5 ± 3 cmH2O between 1998 and 2010 and that lower PEEP levels were independently associated with the occurrence of ICU-acquired pneumonia [49]. Thus, although no study has specifically investigated this issue, a PEEP level between 4 and 8 cmH20 [47], or higher in patients with acute respiratory distress syndrome [50], seems to be rationale. To summarize, it seems to be reasonable to consider protective lung ventilation in such patients who are exposed to a marked inflammatory response.

Hypoxemia and hypercapnia should be strictly controlled, since both may contribute to secondary brain injury, even in patients receiving ECLS [51]. However, the role of oxygenation remains still debated: the results of an exploratory post-hoc substudy of the Target Temperature Management (TTM) trial suggested that hyperoxemia and hypoxemia were not associated with poor neurological outcome and increase in biomarker of brain injury [52], whereas some retrospective and/or meta-analysis of experimental and clinical studies found that hyperoxia could be linked to poor neurological outcome [53] through oxidative stress and potential direct pulmonary and cardiovascular toxicity of oxygen [54]. Finally, preliminary experimental [55, 56] and human [57, 58] studies might suggest a potential interest of hyperbaric oxygenation as a curative treatment of reperfusion injury, with a decrease in neuronal death [55, 56] and an improvement of neurological outcomes [55–57] or cognitive functions [58] after CA not related to carbon monoxide poisoning or gas embolism.

In the multicenter and randomized COMACARE study, targeting a low-normal or a normal-high range in partial pressure of arterial carbon dioxide (PaCO_2_) and oxygen (PaO_2_) during the first 36 h after ICU admission did not affect NSE serum level [59]. However, high-normal PaCO_2_ (5.8–6.0 kPa) and moderate hyperoxia (PaO_2_: 20–25 kPa) resulted in better cerebral oxygenation [59]. Another large multicenter and randomized trial comparing normocapnia and mild hypercapnia in patients after OHCA is still ongoing (NCT03114033).

At that time, current guidelines recommend to target normoxia and normocapnia during the first 72 h [47].

#### Targeted temperature management

Targeted temperature management (TTM) is currently recommended in patients after OHCA with initial shockable rhythm who remain comatose after ROSC and is suggested in patients after OHCA with non-shockable rhythm or after in-hospital CA with any initial rhythm who remain comatose after ROSC, for at least 24 h [47, 60, 61]. TTM should be started immediately at ICU admission [62]. However, the optimal target temperature [63, 64], the optimal duration [65] of TTM as well as the cooling procedures [66–68] are still matter of debate.

Beyond neuroprotective effects [69], TTM might also have cardioprotective effects, especially in patients experiencing post-resuscitation myocardial dysfunction [20]. Currently, there is no sufficient data to contraindicate TTM in patients with post-resuscitation shock. However, when TTM is used, there are some arguments that suggest avoiding lowest temperature targets. In a sub-study of the TTM trial, TTM at 33 °C was associated with more frequent hemodynamic alterations (decreased heart rate, elevated levels of lactate, and need for increased vasopressor support) compared with TTM at 36 °C [70].

### Specific treatments

#### Coronary reperfusion

There is a large consensus for considering acute coronary disease as a frequent cause of CA in adult patients [71, 72]. By analogy with the management of other acute coronary syndromes, the most common strategy is to perform a coronary angiogram (CAG) as soon as possible, since many observational studies reported a significant association between early percutaneous coronary intervention and improved outcome after OHCA [73–75]. Current guidelines argue for a large use of early CAG in these patients [47, 76]. Once the interest of percutaneous coronary intervention in CA of ischemic cause is universally acknowledged, there are several unsolved issues. Among these issues, selection of the best candidates and optimal timing for CAG are the most debated. Regarding the indication, the decision for early CAG should be based on a panel of arguments encompassing previous medical history, warning symptoms before arrest, initial cardiac rhythm, electrocardiographic pattern after ROSC, and biomarkers if available. In addition, recent retrospective data highlight the interest of focusing coronary interventions for patients with preserved neurological status [77]. Regarding the timing, there is a consensus for early CAG (i.e., as soon as possible after hospital arrival) in ST-elevation myocardial infarction (STEMI) patients with preserved neurological prognosis, since this “scoop and run” strategy offers the benefit of both immediate diagnosis and treatment and may avoid secondary cardio-circulatory deterioration related to untreated coronary occlusion [47]. By contrast, a “wait and see” strategy (delayed CAG) may be proposed in patients without evidence of STEMI [78]. Thus, Lemkes and colleagues showed in a multicenter and randomized controlled trial that the survival of patients who had CA without signs of STEMI was similar regardless the timing of CAG. In addition, a delayed strategy avoided a significant number of useless CAG [78]. These two strategies (early versus delayed CAG in non-STEMI patients) are currently evaluated in several ongoing studies (DISCO NCT02309151, COUPE NCT02641626, TOMAHAWK NCT02750462, PEARL NCT02387398, NCT02587494, EMERGE NCT02876458), which should be helpful for establishing future guidelines.

#### Extracorporeal life support

Mechanical circulatory support can be necessary in the most severe forms of post-resuscitation shock, when the neurological prognosis is assumed to be favourable. Several technics have been proposed, such as Impella [79], or intra-aortic balloon pump [80]. However, the post-resuscitation myocardial dysfunction can be very severe and global [10, 20] with unpredictable severity, up to refractory cardiogenic shock. For these reasons, veno-arterial extra-corporeal membrane oxygenation is the most commonly employed ECLS technic in the post-resuscitation shock [81].

The main issue is to identify the most suitable patients with post-resuscitation shock eligible for ECLS. Bascom and colleagues have proposed to use the “CREST score” (Table 1) for early identification of patients carrying the highest risk of circulatory-related death after CA, who could, therefore, be elective candidates for ECLS [82]. It has also been shown that in patients with post-resuscitation shock treated by ECLS, admission SOFA score < 14, initial shockable rhythm and international normalized ratio < 2.4 as well as initial arterial pH (odds ratio = 1.7 per 0.1 increase) and implantation of ECLS later than 24 h after ROSC were associated with survival and thus could be useful triage tools in such patients [83, 84]. Interestingly, 25–28% of these ECLS patients survived to hospital discharge with favourable neurological and long-term outcome [83, 84], supporting the use of ECLS in carefully selected patients with post-resuscitation shock [85].

**Table 1:** Summarize of the different scores that can be used to select eligible patients with post-resuscitation shock to extracorporeal life support

Scores	Points			
***Assessment of risk of circulatory-related death***				
*CREST score*				
History of coronary artery disease	1			
Non-shockable rhythm	1			
LVEF at time of admission < 30%	1			
Shock at presentation	1			
Ischemic time > 25 min	1			
***Assessment of neurological prognosis***				
*CAHP score*				
Age	1.1 × (age − 10)			
Setting	0 if public setting and 24 if home			
Initial Rhythm	0 if shockable and 27 if non-shockable			
Collapse-BLS duration (min)	2.8 × duration			
BLS-ROSC duration (min)	0.8 × duration			
pH	585–77 × pH			
Epinephrine dose during ressuscitation (total)	0 if 0 mg and 27 if 1 or 2 mg			
*OHCA score*				
Ventricular fibrillation or tachycardia	− 13 if the initial recorded rhythm is VF or ventricular tachycardia + 6 × ln (no-flow interval) + 9 × ln (low-flow interval) − 1434/serum creatinine + 10 × ln (arterial lactate)			
No-flow interval (min)				
Low-flow interval (min)				
Serum creatinine (µmol/L)				
Lactate (mmol/L)				
*CAST score*				
	0	1	2	3
Initial rhythm	Shockable	Non-shockable	–	–
Witness/ROSC time (min)	< 20 min	≥ 20 min	No witness	–
pH	≥ 7.31	7.16–7.30	7.01–7.15	≤ 7.00
Lactate (mmol/L)	≤ 5.0	5.1–10.0	10.1–14.0	≥ 14.1
Motor component of Glasgow coma scale	≥ 2	1	–	–
Gray matter attenuation to white matter attenuation ratio	≥ 1.201	1.151–1.200	≤ 1.150	–
Albumin (g/dL)	≥ 3.6	3.1–3.5	≤ 3.0	–
Hemoglobin (g/dL)	≥ 13.1	11.1–13.0		≤ 11.0

For the CREST score, ischemic time was defined as estimated time from cardiac arrest to return of spontaneous circulation

*LVEF* left ventricular ejection fraction, *BLS* basic life support, *Ln* natural logarithm, *ROSC* return of spontaneous circulation

Beyond hemodynamic severity, the neurological prognosis should be also considered before the decision of ECLS in patients with post-resuscitation shock. Several scores have been proposed to assess neurological prognosis after OHCA [77, 86–88] (Table 1) and could be useful to guide the therapeutic strategy in patients experiencing CA [77].

To summarize, ECLS should be considered as a bridge-to-recovery only in the most severe patients with post-resuscitation shock with preserved neurological status, assessed by some selected prognostic factors or specific scores (Table 1).

### Epurative treatments

Regards to the important release of inflammatory mediators and cytokines in patients with post-resuscitation shock, animal studies have suggested that an early blood removal of inflammatory mediators could be associated with an improvement in hemodynamics and outcome [89, 90]. However, inflammatory mediators are relatively large molecules and it is thus unlikely that the membranes used for conventional renal replacement therapies allow one to achieve high level of cytokines removal, conversely to other alternative extracorporeal blood purification therapies. In this regard, Laurent and colleagues prospectively assessed the effects of high-volume hemofiltration, a technique known to allow a better removal of cytokines, in consecutive patients with post-resuscitation shock [91]. Compared to conventional renal replacement therapy, the use of high-volume hemofiltration was associated with a better survival, while there was no significant effect on the cytokines levels [91]. Such a lack of effect on cytokines removal on hemodynamics was confirmed in more recent animal [92] or human studies [93] pleading against cytokine removal in ischemia–reperfusion syndrome, whatever the epurative technique used.

This lack of effect may be explained by several mechanisms: (1) the lower cytokines level at ICU admission (compared to patients with septic shock) [22], (2) the quick decrease in cytokines level after the initial and transient increase at the time of ROSC [91, 92], and/or (3) the membrane fouling, resulting in a progressive better removal of molecules with lower molecular weights despite the use of high cut-offs membranes.

Thus, given the potential prolonged effects on hemodynamics of the inflammatory mediators and cytokines despite their very short half-life, future therapies should rather focus on agents able to block the inflammatory cascade following the release of inflammatory mediators and cytokines, than to epurative treatments.

#### Steroids

The use of steroids in patients with post-resuscitation shock is still debated despite the evidence for the hormonal dysfunction. Although beneficial effects of glucocorticoids administration during cardiopulmonary resuscitation have been suggested by retrospective or pilot studies [94, 95], only a few studies have focused on the impact of corticosteroids administration in successfully resuscitated patients. In a randomized controlled trial by Mentzelopoulos and colleagues [96] comparing a strategy combining vasopressin, methylprednisolone and epinephrine versus epinephrine alone, patients who were successfully resuscitated received either a stress-dose of hydrocortisone (300 mg daily for 7 days) or saline. Interestingly, the administration of hydrocortisone (at least one dose) improved survival to hospital discharge with favorable neurological status, suggesting a potential benefit of steroids. Nevertheless, because multiple interventions were concomitantly used, it is difficult to affirm the effect of hydrocortisone itself on outcome. More recently, Donnino et al. evaluated the interest of hydrocortisone administration (300 mg daily for 7 days) in a randomized, double-blind, placebo-controlled trial including 50 patients with refractory post-resuscitation shock [97]. Compared to placebo administration, no beneficial effect of hydrocortisone on mortality, time to shock reversal or shock reversal, or neurological outcome was observed. However, patients with documented adrenal insufficiency who received hydrocortisone tended to achieve shock reversal more frequently than those receiving placebo [97].

In a recent work, Tsai et al. have shown in a retrospective analysis of the Taiwan National Health Insurance Research Database that the administration of steroids during the post-CA period was associated with better survival to hospital discharge and 1-year survival in patients receiving low-dose of steroids (< 50 mg daily equivalent prednisone, i.e., about 200 mg/day of hydrocortisone) only [98]. Conversely, higher doses of steroids could even be associated with worse outcomes than in patients not receiving steroids [98]. Despite the retrospective design of the study and the fact that these results come from health insurance databases (with many potential confounders), the administration of low-dose of hydrocortisone in patients with post-resuscitation shock might be of interest, especially in patients with associated relative adrenal insufficiency. Further randomized trials are needed to clarify the potential interest of steroids in patients with post- resuscitation shock.

#### New perspectives

Although the impairment of the hypothalamic release of arginine-vasopressin contributes to the vasoplegia of the post-resuscitation shock [33], only few data are available regarding the potential interest of vasopressin administration in patients with post-resuscitation shock [96, 99] and only one study investigated the potential isolated effect of vasopressin administration in such patients [99]. Thus, Mayr and colleagues retrospectively reported the hemodynamic effects of arginine-vasopressin administration in 23 patients with post-resuscitation shock unresponsive to hemodynamic therapy including fluids, norepinephrine, epinephrine and milrinone. Arginine-vasopressin administration significantly increased MAP and decreased the catecholamines requirement and blood lactate level [99]. Despite the retrospective design of the study and the small number of patients, these results should encourage to further evaluate the effects of arginine-vasopressin, possibly in combination with low-dose of hydrocortisone, in an attempt to achieve the “hormonal healing” in patients with post- resuscitation shock.

Finally, to further investigate the gut dysfunction in post-CA patients, an ongoing study (ENTRACT study, NCT02349074) aims at determining the incidence of upper gastro-intestinal tract ischemia by performing systematic gastroscopy in all patients experiencing CA.

## Conclusion

The post-resuscitation shock results from the whole-body ischemia–reperfusion process provoked by the sequence of circulatory arrest, resuscitation manoeuvers and return of spontaneous circulation. It is an early and transient complication of the post-resuscitation phase, which frequently results in multiple-organ failure and high mortality. Its pathophysiology is complex and multifactorial, combining a myocardial dysfunction and characteristics common to sepsis, such as vasoplegia, hypovolemia and endothelial dysfunction. Treatment is based on an early and aggressive hemodynamic management, including ECLS in the most severe patients, associated with coronary reperfusion when needed. Further clinical research is needed in order to explore new therapeutic opportunities regarding inflammatory, hormonal and vascular dysfunction.

## Data Availability

Not applicable.

## Abbreviations

CA: Cardiac arrest
CAG: Coronary angiogram
ECLS: Extracorporeal life support
ICU: Intensive care unit
MAP: Mean arterial pressure
NSE: Neuron-specific enolase
OHCA: Out-of-hospital cardiac arrest
PaCO_2_: Arterial carbon dioxide partial pressure
PaO_2_: Arterial oxygen partial pressure
PEEP: Positive end-expiratory pressure
ROSC: Return of spontaneous circulation
TTM: Targeted temperature management
